# The increasing popularity of Peruvian maca (*Lepidium meyenii*) and its potential impacts on sleep and quality of life

**DOI:** 10.1016/j.clinsp.2024.100398

**Published:** 2024-06-05

**Authors:** Vinícius Dokkedal-Silva, Priscila Kalil Morelhão, Sergio Tufik, Monica Levy Andersen

**Affiliations:** Department of Psychobiology, Universidade Federal de São Paulo, São Paulo, SP, Brazil

## Abstract

• Peruvian maca is a popular supplement consumed to increase energy and sexual health.
• Studies report it also has anti-sinflammatory and neuroprotective effects.
• Stimulant properties of this supplement raise questions about its effects on sleep.
• Investigation of possible reinforcing characteristics is also recommended.

Dear Editor,

Peruvian maca (*Lepidium meyenii*) is a plant native to the Andes region in South America and has been used as a traditional medicine for centuries by local people. In recent years, there has been a surge of interest in the plant and its potential uses. Reports indicate that maca is a useful natural provider of energy and a nutritious vegetable.^1^ There have been a number of studies on maca being published, diversifying the current body of knowledge about this herb.

A multitude of qualities have been attributed to Peruvian maca and its different products, such as flour and gelatinized powder. *L. meyenii* is suggested to have strong antioxidant, anti-inflammatory and immunomodulatory properties.^1^^,^^2^ In another instance, analgesic action was identified in results from animal experiments.^3^ It has been reported as being able to reduce osteoporosis progression in older people.^4^ One of its main selling points has been as a supplement that can improve fertility and libido, with one study reporting an increase in sexual desire following daily treatment for 8 weeks with gelatinized maca. It is noteworthy that this change was not linked to any significant rise in testosterone and estradiol levels.^5^ In a review, sperm quality was found to be improved both in infertile men (in which increases in sperm motility were the main significant finding) and in healthy volunteers (improvements in sperm count and morphology) after the use of maca.^6^ Evidence has also implied that it could be an important ally in the treatment of perimenopausal symptoms, including insomnia and other sleep complaints.^7^

Although there is an increasing body of evidence promoting the benefits of maca and its products, several questions still remain to be fully answered, including those in regard to the effectiveness and safety of this plant. Despite a number of communications on the beneficial action of maca on menopausal symptoms, most were based on anecdotal evidence or small-scale samples. The meta-analysis by Lee et al.^8^ did not identify a significant amelioration of menopausal symptoms. Afterward, although the same authors in their 2016 meta-analysis found a number of studies reporting that treatment with maca was beneficial to sperm parameters in men, only 5 of these efforts were included, of which one was an abstract, 2 were uncontrolled observational studies and 1 was an unpublished RCT.^6^ It must be added that the authors pointed out that all the initiatives were supported by maca manufacturers, possibly introducing a degree of bias.^6^ Another point to be made in this regard is the lack of studies covering sleep effects in a broader population scope. As maca has been commercialized as a supplement to be used regardless of age and commonly due to its boosting effects on testosterone, its influence on sleep in younger people and in males is also of utmost importance. Another pressing necessity is comparing maca with other known natural sleep aids, both in clinical and in pre-clinical studies. To the best of our knowledge, no study comparing maca with pre-existing strategies has been performed.

While maca is advertised as a natural, safe stimulant, there is still a lack of empirical evidence of its alleged safety.^6^ Stimulant substances are known to frequently bring about unwanted side effects, such as mood alterations and severe sleep impairment. No controlled study has been performed to evaluate these possibilities. Long-term effects of maca have been studied before, but the scope of studies was limited to spermatogenic effects. Administration of maca for prolonged periods was linked to improvement in sperm production in the long term in pre-clinical models.^9^^,^^10^ Gonzales and colleagues did not report side effects from long-term administration of three cultivars of maca to mice.^10^ Nevertheless, these findings constitute one of the few initiatives to map long-term maca effects and its impact on other health parameters remains to be reported.

It has been hypothesized that maca consumption may cause similar behaviors to recreational stimulants due to having among its chemical compounds the carboxylic acid MTCA. This molecule is suspected to cause substance craving, a common trait observed in those with substance use disorders.^11^ As pointed out by Piacente et al.^12^ MTCA has been investigated for numerous central action effects. It has been shown to inhibit monoamine oxidases, allowing for the amplified effect of psychotropic substances in the ayahuasca preparation.^13^ This possibility deserves special focus, as a link with nicotine addiction and possible reinforcing properties of chocolate and cocoa has been suggested.^14^ Limited evidence suggests it is also a role in the etiology of alcoholism,^15^ but further research on this field is scarce until the present day. Besides, some attention needs to be given to the fact this molecule can be a comutagen or a precursor of mutagenic compounds, which has elicited caution in its approval in some markets.^12^ A clear direction in which the role MTCA functions is still unfortunately scarce, constituting an additional reason for extensive research on its central action to be performed.

Although the data exposition here is brief, it evidences the necessity of critical research on not only benefits, but also the risks of Peruvian maca. It is suggested an expansion of assessments for this herbal preparation, covering other populations besides peri- and postmenopausal women, with studies that investigate sleep quality, stimulant properties and possible reinforcing effects. In this sense, further evaluation of the MTCA is also a priority in itself, due to the many suggested effects but the scarcity of concrete evidence on its role. While the qualities presented by *L.meyenni* must not be ignored, a complete profiling of its nature and extent of its positive and negative aspects will ensure safer consumption, a conscious approach to its use, and the preservation of the quality of life of its users.

## Funding

Our studies are supported by grants from the Associação Fundo de Incentivo à Pesquisa (AFIP). MLA and ST are Conselho Nacional de Desenvolvimento Científico e Tecnológico (CNPq) fellowship recipients.

## CRediT authorship contribution statement

**Vinícius Dokkedal-Silva:** Conceptualization, Writing – original draft, Writing – review & editing. **Priscila Kalil Morelhão:** Writing – review & editing. **Sergio Tufik:** Resources, Funding acquisition. **Monica Levy Andersen:** Supervision, Validation, Visualization, Writing – review & editing.

## Conflicts of interest

The authors declare no conflicts of interest.

## Appendix Supplementary materials

Image, application 1
