# Supplementary material for: The increasing popularity of Peruvian maca (Lepidium meyenii) and its potential impacts on sleep and quality of life
Source: Clinics (Sao Paulo). 2024 Jun 5;79:100398. doi: 10.1016/j.clinsp.2024.100398 (PMC11214369; doi:10.1016/j.clinsp.2024.100398)
Supplement: Supplementary file 1 [file mmc1.docx]

The increasing popularity of Peruvian maca (Lepidium meyenii) and its potential impacts on sleep and quality of life - Highlights

- Peruvian maca is a popular supplement consumed to increase energy and sexual health
- Studies report it also has anti-inflammatory and neuroprotective effects
- Stimulant properties of this supplement raise questions about its effects on sleep
- Investigation of possible reinforcing characteristics is also recommended
